# Parasite epigenetics and immune evasion: lessons from budding yeast

**DOI:** 10.1186/1756-8935-6-40

**Published:** 2013-11-19

**Authors:** Brandon A Wyse, Roxanne Oshidari, Daniel CB Jeffery, Krassimir Y Yankulov

**Affiliations:** 1 Department of Molecular and Cellular Biology, University of Guelph, Guelph, ON N1G 2 W1, Canada

**Keywords:** Antigenic variation, Allelic exclusion, Telomere position effect, Gene silencing, Epigenetic switch

## Abstract

The remarkable ability of many parasites to evade host immunity is the key to their success and pervasiveness. The immune evasion is directly linked to the silencing of the members of extended families of genes that encode for major parasite antigens. At any time only one of these genes is active. Infrequent switches to other members of the gene family help the parasites elude the immune system and cause prolonged maladies. For most pathogens, the detailed mechanisms of gene silencing and switching are poorly understood. On the other hand, studies in the budding yeast *Saccharomyces cerevisiae* have revealed similar mechanisms of gene repression and switching and have provided significant insights into the molecular basis of these phenomena. This information is becoming increasingly relevant to the genetics of the parasites. Here we summarize recent advances in parasite epigenetics and emphasize the similarities between *S. cerevisiae* and pathogens such as *Plasmodium*, *Trypanosoma*, *Candida*, and *Pneumocystis*. We also outline current challenges in the control and the treatment of the diseases caused by these parasites and link them to epigenetics and the wealth of knowledge acquired from budding yeast.

## Review

### Mechanisms of antigenic variation and immune evasion

Many protozoan parasites and pathogenic fungi use antigenic variation as the major strategy to evade the host immune defenses [1-3]. The genomes of these species harbor extended families of genes that encode closely related surface proteins (Table 1). In any given cell, all but one gene of these families are repressed by compact chromatin structures. These structures are refractory to transcription and are epigenetically transmitted to daughter cells. Occasional and reversible switches to a different active gene confer antigenic variation. These ever-changing ‘cloaks of invisibility’ enable the pathogens to persist in the hosts with devastating efficiency [1,4].

**Table 1:** Varying genes and known mechanisms of variation in different pathogens

**Species**	**Varying genes (number of genes given in parentheses)**	**Mechanisms involved in gene variation**	**Reference(s)**
*Trypanosoma brucei*	*VSG* (>1,000)	Epigenetic switches, DNA recombination	[3]
*Pneumocystis carinii*	*MSG* (160)	DNA recombination	[5,6]
*Plasmodium falciparum*	*VAR* (60), *rifin* (150 to 200), and *stevor* (30 to 35)	Epigenetic switches	[7-10]
*Candida glabrata*	*EPA* (23)	Epigenetic switches	[2,11]
*Giardia lamblia*	*VSP* (220)	RNA interference	[12-14]
*Saccharomyces cerevisiae*	Various subtelomeric genes	Epigenetic switches	[15,16]
*S. cerevisiae*	Mating type loci (*HMRα* and *HMLα*)	DNA recombination	[17]

### Silent ‘donor’ genes in Trypanosoma and Pneumocystis

Trypanosomes are bloodstream parasites with a most remarkable ability to evade the immune system and to cause severe diseases such as nagana (*Trypanosoma vivax*, *T. congolense*) and sleeping sickness (*T. brucei*) [3]. These maladies are characterized by extreme fatigue and sleepiness. *T. brucei* is the prevalent pathogen in humans and has become a prototype for antigenic variation. It harbors a massive family of more than 1,000 mostly subtelomeric variant surface glycoprotein (*VSG*) genes and pseudogenes.

**Figure 1:**
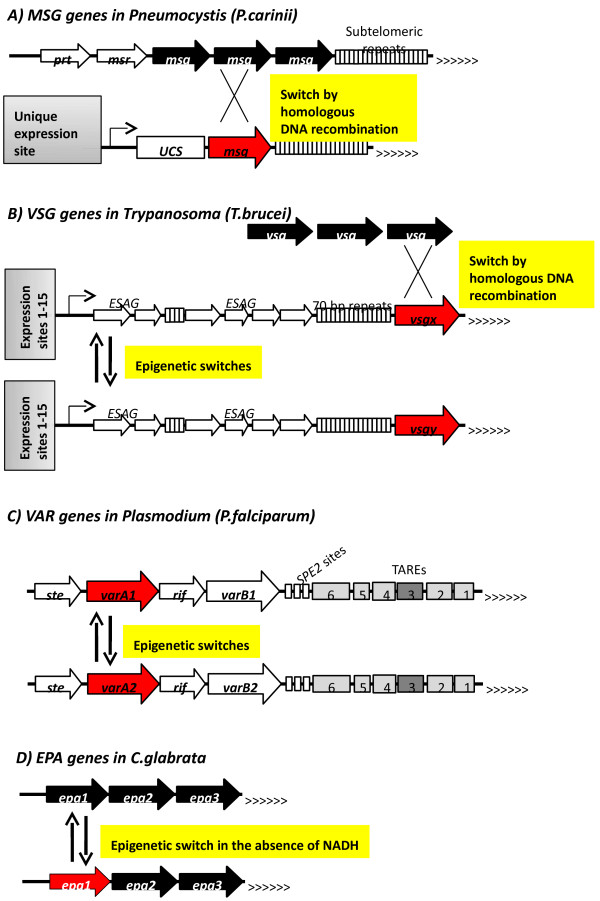
**Schematics of varying genes and major mechanisms of variation in *Pneumocystis carinii*, *Trypanosoma brucei*, *Plasmodium falciparum*, and *Candida glabrata*. (A)** In *P. carinii,* one to three *MSG* gene arrays (frequently flanked by *MSR* or *PRT* genes) are positioned next to a variable number of subtelomeric repeats and the telomeres (depicted by >>>). These genes (black arrows) lack promoters. To be expressed, a single *MSG* (red arrow) is transferred by homologous recombination to a unique expression site that contains an upstream conserved sequence (*UCS*). **(B)** In *T. brucei,* over 1,000 *VSG* gene donor sequences (black arrows) are exchanged by homologous recombination to one of fifteen expression sites. The *VSG* genes at these sites (red arrows) are adjacent to the telomeres and flanked by multiple 70 bp repeats. Several expression site-associated genes (*ESAG*s, open arrows) are distal to the telomere. All these and *VSG* are expressed in the direction of the telomere by one promoter (angled arrow). Only one of the fifteen expression sites is active at a time. Infrequent epigenetic switches of this site confers allelic exclusion and antigenic variation. **(C)** In *P. falciparum,* 60 *VAR* genes are positioned in tandem close to the telomeres or at interchromosomal locations (not shown). *VAR*_*A*_ (red arrow) point towards the telomere, while *VAR*_*B*_ (white arrow) point away. Six telomere-associated repeat elements *(TARE*) and several 12-base *SPE* sites (bind *P. falciparum* SPE2 interacting protein 2, PfSIP2) are located between *VAR* genes and the telomere. *RIF* and *STE* genes are frequently found in the vicinity of *VAR* genes. Only one *VAR* gene is expressed at a time. Switches between expressed *VAR* genes confer allelic exclusion and antigenic variation. **(D)** In *C. glabrata, EPA* genes are organized in arrays and are the last protein encoding genes before the telomere. In the example shown in the figure, *EPA3* and *EPA2* are mostly silenced, while *EPA1* is expressed. *ESAG*, expression site-associated gene; PfSIP2, *P. falciparum* SPE2 interacting protein 2.

The fungi of the *Pneumocystis* family reside within the mammalian lungs and normally cause no symptoms, but can lead to serious infections in immunocompromised individuals and in HIV-infected patients [5,6]. Antigenic variation in *Pneumocystis* is produced by about 160 major surface glycoprotein (*MSG*) genes. Many of these are the last protein encoding genes at the telomeres of the 17 chromosomes [18,19] (Figure 1A). Interestingly, the interchromosomal *MSG* genes or pseudogenes are also surrounded by telomeric repeats [18].

The only active *VSG* in *T. brucei* is expressed from one of 15 dedicated expression sites, while the active *MSG* in *P. carinii* is expressed from one unique expression site [4,5] (Figure 1A,B). These expression sites are adjacent to a telomere and contain gene promoters plus other regulatory elements. The pools of silent intact *VSG* and *MSG* genes plus pseudogenes and other *VSG* and *MSG* homologous sequences serve as a depot of donor elements that are translocated to the expression sites via DNA recombination [5,20] (Figure 1A,B). It is not known how the frequency of these recombination events is controlled [3]. However, it seems apparent that the silencing of the *VSGs* in *T. brucei* is accomplished by epigenetic means [21,22]. Strong support for this idea is offered by the observations that the knockdowns of key heterochromatin regulators *(SIR2*, *RAP1*, *DOT1A)* leads to their derepression [21,23,24]. No genetic evidence is available from *Pneumocystis*. It is noteworthy that a similar constitutive repression of ‘donor’ genes and their translocation to an active site governs the switching of the mating type in *S. cerevisiae*[17]. These parallels are extensively covered [3-5] and will not be reviewed here.

### Epigenetic switching of subtelomeric genes in Trypanosoma, Plasmodium, and Candida

Of the 15 *VSG* expression sites in *T. brucei*, only one is active at a time. Early during the infection, the *VSG* switching is conducted mostly by rapid epigenetic on-off transitions between these expression sites (Figure 1B). Later on, the switching involves both epigenetic and DNA recombination events [3,4]. It is not known how the expression from a single site is achieved.

Different species of *Plasmodium* cause malaria by invading red blood cells in a wide variety of organisms. The pathogens undergo a complex life cycle that involves transmission by mosquitoes and a latency period in the livers of the hosts [7,8]. *P. falciparum*, one of the most extensively studied malaria pathogens, is chosen as a paradigm for gene variation by epigenetic switches. During blood-stage infection this pathogen expresses alternative forms of the immunodominant antigen *P. falciparum* erythrocyte membrane protein 1 (PfEMP1). The expressed PfEMP1 is trafficked by specialized vesicular structures and then displayed on the surface of the infected erythrocytes [7]. Using PfEMP1 as an adherent, the infected erythrocyte is sequestered to the vascular walls to contribute to the severe symptoms of malaria. PfEMP1 is encoded by a limited family of 60 *VAR* genes, which are positioned in the subtelomeric regions of the chromosomes and in several interchromosomal clusters. An elaborate and poorly understood mechanism of coordinated silencing of the *VAR* genes is combined with rare epigenetic switches to other variants to confer an ever-shifting antigenic makeup (Figure 1C). This strategy is sufficient to minimize recognition of PfEMP1 by the immune response [7] and, at the same time, prevents the exhaustion of the reservoir of *VAR* genes [25-27]. Other families of surface proteins (*rifin*, *stevor*, *PfMC-2TM*) also contribute to antigenic variation, but PfEMP1 is believed to be the critical driving force of the immune evasion [7,9]. Besides epigenetic switches, there is solid evidence for frequent gene conversions between *VAR* genes [9]. While these contribute to the diversification of the gene family, such events do not directly contribute to the switching of the PfEMP1 surface antigens.

The coordinated silencing of all but one *VAR* gene is the crux of prolonged malaria infections and the slowly developing and incomplete immunity to the pathogen. Consequently, the factors that contribute to antigenic variation in *P. falciparum* have been extensively studied [7,9,25,28]. While a significant body of information has been acquired, the mechanisms of *VAR* silencing remain unknown [9,26,27,29-31]. For example, it is not clear what kind of *cis*-elements serve as *VAR* silencers. The introns [32] and a conserved region upstream of the *VAR* promoters [30,33,34] have been proposed to act as silencing elements, but later on the significance of the intron has been debated [7,30]. The pairing of *VAR* genes has also been proposed to contribute to repression, but the mechanistic details are yet to be elucidated [30,34]. However, it has been conclusively shown that histone acetylation, histone methylation, and the propagation of heterochromatin away from the telomeres control the *VAR* genes [35-39]. Another line of evidence suggests that the tethering of *VAR* genes to poorly characterized subdomains in the nuclear periphery could be critical for both their repression and switching [31,40,41]. In addition, there is widespread expression of long non-coding RNAs in blood-stage *P. falciparum*[42-45], but no conclusive evidence for the regulation of *VAR* genes by such RNAs has been obtained [46-48].

*Candida glabrata* is an opportunistic parasite that causes prolonged urinal tract infections [6]. The key event in these infections is the adhesion of *C. glabrata* to host epithelial cells via epithelial adhesin (*EPA*) genes. Antigenic variation in this species is conferred by 23 *EPA* genes, which are positioned in the subtelomeric regions (Figure 1D) and are repressed by the NADH-dependent histone deacetylase *SIR2*[49,50]. Interestingly, *C. glabrata* is a nicotinic acid auxotroph. It is believed that the repression of *EPA* genes relies on the NADH provided by the host. Once the parasite moves to the urinary tract (there is very little nicotinic acid in the urine) the activity of Sir2p diminishes and the *EPAs*, and *EPA1* in particular, are expressed [2,11].

### RNA interference in Giardia

*Giardia lamblia* is a human intestinal pathogen that causes mild to severe diarrhea. Infection is transmitted by ingestion of cysts followed by the attachment of *Giardia* to the intestinal cells via variant-specific surface protein (*VSP*) genes. *G. lamblia* displays distinctive antigen variation through changes in the expression of about 220 *VSP* genes [51]. However, there are notable distinctions in the control of variation between *Giardia* and the previously described species. First, *VSP* genes are not clustered in the subtelomeric regions. Second, it has been suggested that multiple *VSP* genes are expressed and then all but one of the *VSP* mRNAs are repressed by an elaborate endogenous RNAi system that remains to be fully characterized [12-14]. No DNA methylation has been demonstrated in this organism [12].

### Telomere position effect (TPE) in S. cerevisiae: similarities in pathogens

**Figure 2:**
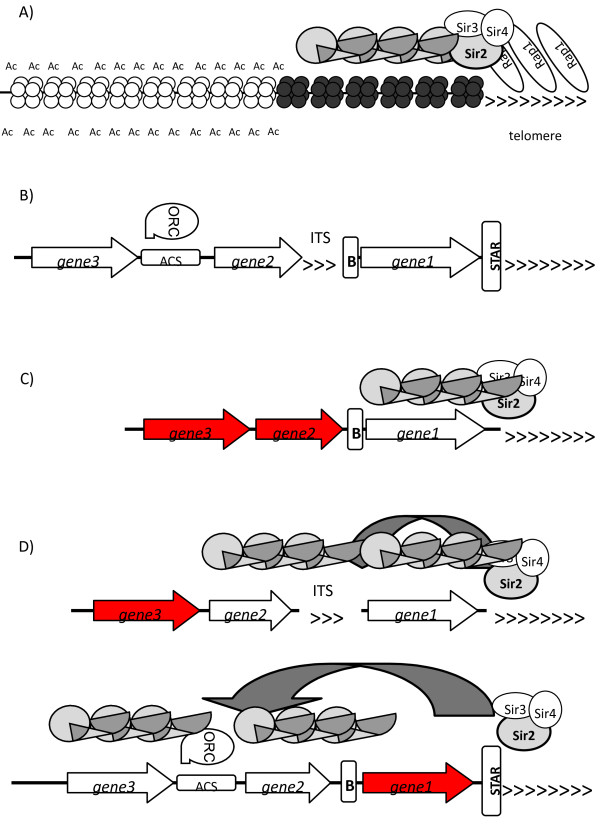
**Subtelomeric gene silencing in *Saccharomyces cerevisiae*. (A)** Spreading of histone deacetylation away from the telomere. Rap1 proteins associate with the telomere repeats and recruit Sir2/Sir3/Sir4 proteins. Sir2p is an enzyme that deacetylates the histones in the adjacent nucleosome. More Sir2/Sir3/Sir4 proteins are recruited by the now deacetylated nucleosome (dark octamer) to eventually spread histone deacetylation to the next nucleosome (depicted by the curved arrow above the nucleosomes). Histone deacetylation and silent information regulator (SIR) proteins can spread several kilobases away from the telomeres. **(B)** Subtelomeric *cis*-elements in *S. cerevisiae.* Repetitive *core X* and *Y’* elements contain dormant origins of DNA replication (*ACS*, it binds origin recognition complex, ORC), internal telomeric sequences (*ITS*, they bind Rap1 proteins), chromatin boundaries (depicted by ***B***, and subtelomeric anti-silencing regions (*STARs*). **(C)** Chromatin boundaries restrict the spreading of histone deacetylation and prevent the silencing of telomere-distal genes (red arrows). **(D)***ITS* and *ACS* are protosilencers, which extend the spreading of SIR proteins or confer telomere-dependent silencing of genes (white arrows) beyond an active subtelomeric gene (red arrow). A hypothetical *STAR* and a chromatin boundary contribute to the maintenance of the active gene. ORC, origin recognition complex; SIR, silent information regulator.

Allelic exclusion and variation is the crux in the infections by the above pathogens. However, the mechanisms of gene silencing and switching are not so well understood. On the other hand, a similar (but not identical) phenomenon (referred to as telomere position effect, TPE) in the innocent budding yeasts is significantly better characterized [15,52]. Briefly, silencing is mediated by compact heterochromatin, which is re-established after each cell division. Rare conversions between the silent and active states confer a quasi-stable pattern of epigenetically controlled gene expression in the vicinity of the telomeres [53]. TPE has been extensively reviewed [15,53,54] and is schematically presented in Figure 2A.

Unlike many higher eukaryotes, DNA methylation plays no role in the repression of subtelomeric genes in *S. cerevisiae.* In addition, while long non-coding RNA (called telomeric repeat-containing RNA, TERRA) is produced by the yeast subtelomeric DNA, a role of this RNA in gene silencing and switching has not been established [55]. These peculiarities have rendered this model organism somewhat irrelevant to epigenetics in higher eukaryotes. However, they make it quite relevant to the parasites that were discussed earlier. While budding yeasts cannot represent the whole variety of mechanisms for antigenic variation, some noteworthy similarities do exist. In *Plasmodium*, *Candida*, *Trypanosoma*, and *Pneumocystis*, the gene families that contribute to antigenic variation are mostly or exclusively located in the subtelomeric regions of the chromosomes. The repression of these subtelomeric genes is highly dependent on histone modifications [23,24,39,49,51,56-58], whereas in other eukaryotes the formation and maintenance of heterochromatin is more complex and involves additional levels of regulation. For example, similarly to *S. cerevisiae* and in contrast to higher eukaryotes, DNA methylation and RNAi do not seem to contribute to the silencing of the variance genes [7,15]*.*

In *S. cerevisiae* and *P. falciparum* the silenced variation loci cluster in the nuclear periphery [8,15,59]. In *S. cerevisiae*, the relocation of these loci leads to derepression [59]. In *P. falciparum*, relocation to another ‘active’ domain, still in the nuclear periphery, is believed to contribute to the switching and the activation of *VAR* genes [7,33].

In *S. cerevisiae*, *P. falciparum*, and possibly other pathogens, the epigenetic silencing of genes needs a passage through the S phase [32,53], but it is not clear if DNA replication itself is the required process [60].

### Shared strategies of antigenic variation and the means to combat them

In order to implement antigenic variation, the parasites must execute three distinct tasks. First, they need to selectively and exclusively activate one gene of the family at a time. Second, they need to effectively repress all but one of the genes of the family. Third, they need to switch the active gene at a frequency that runs ahead of the building immune response but does not exhaust the repertoire of the gene family.

### Selective expression of one gene

The key question, how to selectively express one and only one gene of the extended family, remains unanswered. Ostensibly, the means must be coupled to the repression of all other genes, but how a gene is singled out is a persisting mystery. A common theme in the studies in *Pneumocystis* and *Trypanosoma* is the existence of expression sites [3,7]. In *Pneumocystis* the single expression site determines the expressed variant. This situation calls for regulated DNA recombination events at a frequency that will not jeopardize the pool of *MSG* genes. The same applies to the *VSG* genes in trypanosomes except that the situation there is complicated by the multiplicity of expression sites and their exchange through *bona fide* epigenetic means. A clue from *S. cerevisiae* suggests that the expressed site in trypanosomes could be related to the position of the active locus in the nucleus. In *S. cerevisiae* the telomeres cluster in several compartments in the nuclear periphery. Upon translocation to the nucleoplasm the telomeric genes lose repression [59]. A few proteins, including the *Ku* antigen and the nuclear pore components, contribute to this peripheral clustering [59,61]. A similar clustering of the inactive *VAR* genes in the nuclear periphery is apparent in *Plasmodium*, but the active *VAR* gene remains in the nuclear periphery slightly away from the repressed cluster [7,8]. These observations are consistent with the idea that the sub-nuclear localization of the *VAR* or *VSG* loci is linked to the expression of these genes. However, it is not clear if this differential localization is the cause or the consequence of the switch from silenced to active state.

Another clue from budding yeasts points to the fine architecture of subtelomeric DNA as combined with the balance of transcriptional activators and repressors. In *S. cerevisiae*, subtelomeric DNA consists of conserved *core X* and *Y’* elements and harbors degenerate internal telomeric repeats (*ITS*), silent origins of DNA replication (*ACS*), and isolated binding sites for Rap1p (Figure 2B). These act as protosilencers and relay the spreading of SIR proteins away from the chromosome ends (Figure 2D) [62]. Additional complexity is provided by subtelomeric anti-silencing regions (*STARs*) and chromatin boundaries (Figure 2C) [63-65]. Likewise, the folding of the telomere and the establishment of t-loops and G-quadruplexes also contributes to complexity [63,66,67]. In this vein, G-quadruplex structures have been recently characterized in *P. falciparum*[68], while protosilencers have been conclusively identified in *C. glabrata*[69,70]. The *SPE* sites and the subtelomeric *TARE3* in *P. falciparum* (Figure 1C) have also displayed properties consistent with protosilencing or boundary activities [45,71]. It is therefore tempting to speculate that assemblies of *cis*-elements similar to these in *S. cerevisiae* also exist in parasites.

It has been shown that in *S. cerevisiae*, the protosilencers, *STARs*, and chromatin boundaries can confer isolated expression of a gene imbedded in a heterochromatic region (Figure 2D) [64,66]. At the same time, studies in *S. cerevisiae* and *D. melanogaster* have shown that the abundance/strength of transcriptional activators counteract the silencing of target genes [72-74]. In *S. cerevisiae*, it has been demonstrated that overexpression of the *trans*-activator Ppr1p antagonizes the silencing of a telomeric *URA3* reporter and that progression through S phase was necessary for the establishment of the active state [73]. It is conceivable that in parasites a similar mechanism of silent to active transition could exist. For example, an increase of variance gene-specific transcriptional activators and/or *STAR* binding factors accompanied by a passage through S phase could destabilize the repression of all genes in the family and predispose them to a conversion (see model in Additional file 1). Although the currently expressed gene has the advantage to remain active through epigenetic heritance [75], another gene could compete via the engagement of the chromatin boundaries and the gradual sequestration of limiting gene-specific activators. Reversion to a lower abundance of such activators would reinstate the robust repression of the other variance genes and uphold the conversion. Hence, the interplay between weak *cis*-elements and subtle changes in the abundance of transcription factors could significantly contribute to the elusive mechanism of epigenetic switches. In support, such temporary destabilization and expression of multiple *VAR* genes before a single *VAR* gene is selected has already been observed in *P. falciparum*[30,31,75]. Interestingly, there was an increase in the rate of switching at subtelomeric *VAR* loci as compared to switching at the internal loci. By all means, a closer look at the subtelomeric DNA of these parasites and a search for protosilencers, boundary, and/or anti-silencing elements and factors is warranted.

### Repression of the varying genes

Lessons from budding yeasts have provided a basic framework for the understanding of this process in parasites. The central mechanism of the spreading of deacetylation from the telomeres operates in these and many other eukaryotes (Figure 2A) [15,52]; however, some exceptions need to be mentioned. As in budding yeast, Sir2p is a critical factor for the silencing of the varying genes in *P. falciparum* and *C. glabrata*, but it is not essential for the *VSGs* in *T. brucei* and its role in *P. carinii* is unknown [11,35,40,49,56-58,76]. Similarly, the telomere-binding protein Rap1 is essential for telomeric silencing in *T. brucei* and *C. glabrata*[56], but no evidence for its role in *Plasmodium* or *Pneumocystis* is available. Another feature that appears conserved between parasites and yeasts is the existence of histone variants that are specific to silent and active chromatin. In *S. cerevisiae*, H2A.Z antagonizes telomeric silencing and is enriched at chromatin boundaries [77]. A similar but more complex exchange of histones functions in *P. falciparum* where the unusual H2A.Z/H2B.Z double-variant nucleosomes are prevalent at active genes, but are excluded from silenced *VAR* genes [78]. Histone variants have also been described in *T. brucei*, but their relevance to gene silencing, if any, is not clear [79,80].

The methylation of histones poses even greater uncertainty. Methylation at specific K/R residues is associated with both gene activation and gene repression and is catalyzed by two classes of histone methyltransferases, SET and DOT1 [81]. In *S. cerevisiae*, the trimethylation of H3K79 by Dot1p has long been considered a key event in telomeric silencing [81], while the methylation of H3K36 by Set2p has been continuously linked to active transcription [82]. It was surprising to learn that the methylation of H3K36 by *P. falciparum* variant-silencing SET (PfSETvs) was critical for the repression of *VAR* genes in *P. falciparum*[39]. In *T. brucei*, the deletion of *DOT1B* does not lead to a general derepression, but increases the duration of the epigenetic switch [24]. In summary, although histone methylation at specific residues certainly contributes to gene silencing, significant variations between different parasites and *S. cerevisiae* could be expected.

Where does this notion lead us? It is feasible that the disruption of histone acetylation and methylation will preclude gene silencing and will cause the expression of many if not all of the varying genes. Indeed, it has been shown that the deletion of the homologues of *SIR2*, *DOT1*, or *SET2* can produce pathogens that display multiple antigens [24,39,58]. These mutants, when properly attenuated, can be used for the successful generation of vaccines. Currently, the lack of vaccines is one of the most haunting issues in malaria and sleeping sickness [7,39,83-85]. In this respect, the gained knowledge of gene silencing can deliver a major breakthrough in the prevention of these devastating maladies. It is also conceivable that the drug targeting of the parasite homologues of the Sir2, Rap1, Set2, or Dot1 proteins can be used to combat the infections. To date, inhibitors of histone deacetylases or methyltransferases have shown promise under laboratory conditions [38,86]. However, this approach certainly needs fine-tuning. Because the varying surface antigens are directly linked to morbidity, their potential overexpression could produce ‘super-pathogens’ in the patients and will offset any gain in immunity. The risk of such a possibility has been demonstrated *in vitro* in *C. glabrata*[87].

### Reversible epigenetic switches

An alternative to the risky and harmful overexpression of surface antigens is the reduction in the frequency of epigenetic switches. The rationale is that the immune system would gain ample opportunity to combat and clear the parasite. Unfortunately, the actual mechanisms of epigenetic conversions in *S. cerevisiae* or in the pathogens are not known [52]. While many regulators of TPE have been identified, the majority of them expand or contract the subtelomeric heterochromatin domain [15,88]. Hence, they do not necessarily alter the frequency of switching. Mutations in other regulators produce higher levels of the expression of otherwise silenced reporters [89,90], but it is hard to tell if modest loss of repression or frequent epigenetic conversions have yielded these results.

A decrease in the rate of switching is a reliable criterion for true deregulation, but it is rarely observed. To our knowledge, only one study in parasites *(T. brucei)* has shown such an effect. As mentioned above, in this species the deletion of *DOT1B* retards the epigenetic switch to a point where the cells express two *VSGs* for weeks [24]. Interestingly, in *S. cerevisiae* the trimethylation of H3K79 (it is catalyzed by Dot1p), but not *DOT1* itself, increases the rate of the silencing establishment [91]. Five other studies in *S. cerevisiae* have reported the so-called ‘enhanced memory for heritable transmission’. Two of them have characterized mutations in histone H4, which increase the stability of both the repressed and the active states of subtelomeric reporters [92,93]. Two other papers [94,95] have pointed out that *SIR1* alters the frequency of conversion at the mating type loci. Sir1p binds to dormant origins of DNA replication (these act as silencers of the mating loci) and recruits Sir2p [53]. Recently, we reported that the deletion of *CAC1* reduces the frequency of epigenetic conversions of subtelomeric reporters [96]. Cac1p is a component of chromatin assembly factor I (CAF-I), which travels along with the replication forks and reassembles H3/H4 into nucleosomes on newly synthesized DNA [97]. It seems that both local silencers and the passage of replication forks act to occasionally change the epigenetic state of genes.

It is of particular interest that a replication fork factor contributes to epigenetic conversions. It is well known that the passage of the fork disperses the existing nucleosomes [52]. The subsequent reassembly combined with subtle variations in the abundance of variant gene-specific factors could both bestow the opportunity for a switch, as depicted in Additional file 1. This notion is in tune with the observations in *S. cerevisiae* and *P. falciparum* that the establishment of silencing requires a passage through S phase [7,52]. In a similar vein, a recent study in *P. falciparum* has demonstrated that the repositioning of a gene (*P. falciparum* reticulocyte binding protein-like homologue 4, PfRh4) to an active site in the nuclear periphery is associated with more frequent active to silent epigenetic switching [98]. It is attractive to speculate that these conversions are promoted by the open chromatin environment and that both replication forks and the state of existing nucleosomes determine the frequency of epigenetic switches. At present, CAF-I is the only candidate that could potentially confer reduced switching and non-varying phenotype in the parasites. However, other histone chaperones such as the homologues of the yeast *ASF1*, *Rtt106*, *FACT*, or *HST* should be considered. The exploration of this possibility may generate new drug targets and a truly new class of anti-pathogen drugs.

## Conclusions

Parasites like *Plasmodium* and *Trypanosoma* cause devastating maladies in millions of people and are a leading cause of death in many developing countries. Others such as *Pneumocystis* could be deadly opportunistic agents. They all share a common powerful weapon: antigenic variation. The remote yeast *S. cerevisiae* has provided a paradigm and a framework to study positional effects, which are very relevant to the underlying mechanisms of antigenic variation. In the opinion of the authors, researchers need to turn more often to yeasts for clues on how to disarm such pathogens.

## Abbreviations

ACS: ARS consensus sequence; ARS: Autonomously replicating sequence; CAF-I: Chromatin assembly factor I; DOT1: Disruptor of telomeric silencing 1; EPA: Epithelial adhesin; ESAG: Expression site-associated gene; ITS: Internal telomeric sequences; MSG: Major surface glycoprotein; ORC: Origin recognition complex; PfEMP1: *P. falciparum* erythrocyte membrane protein 1; PfRh4: *P. falciparum* reticulocyte binding protein-like homologue 4; PfSETvs: *P. falciparum* variant-silencing SET; PfSIP2: *P. falciparum* SPE2 interacting protein 2; RAP1: Repressor activator protein-1; RNAi: RNA interference; SIR: Silent information regulator; STAR: Subtelomeric anti-silencing region; TARE: Telomere-associated repeat element; TERRA: Telomeric repeat-containing RNA; TPE: Telomere position effect; UCS: Upstream conserved sequence; VSG: Variant surface glycoprotein; VSP: Variant-specific surface protein.

## Competing interests

The authors declare that they have no competing interests.

## Authors’ contributions

BW, RO, and DJ participated in the review of the literature, in the writing of the manuscript, and in the preparation of the figures. KY conceived and drafted the review and wrote the final version of the manuscript. All authors read and approved the final manuscript.

## Supplementary Material

Additional file 1 **Model for epigenetic conversions driven by subtle fluctuations of activators and/or silencing factors. (1)** All but one of a family of hypothetical varying genes (*VG1* to *VGN*) are maintained in a silenced state. These genes are flanked by a subtelomeric anti-silencing region (*STAR*) and a chromatin boundary (B). **(2)** A subtle increase in the abundance of gene activators (green circle) and/or factors that engage *STAR* (purple circle) and the concomitant passage of replication forks would allow the activators access to the promoters of the *VG* genes and **(3)** would predispose all *VG* loci to derepression. **(4)** Consequently, during the next stage (re-establishment of silencing), the derepressed *VG* genes (*VG2* to *VGN*, pink) will compete with the currently active gene (*VG1*, red). **(5)** During this stage, a decline in the abundance of activators and *STAR*-acting factors would aid in the formation of heterochromatin and the limiting activators would be gradually sequestered to a single locus. There is a high probability that the currently active gene (*VG1*) will be reinstated as the active locus. However, switches to another gene are possible (*VGN*). The likelihood of such switches, depicted by the width of the arrow, represents the frequency of epigenetic conversions. Click here for file
