# Supplementary material for: Parasite epigenetics and immune evasion: lessons from budding yeast
Source: Epigenetics Chromatin. 2013 Nov 19;6:40. doi: 10.1186/1756-8935-6-40 (PMC3843538; doi:10.1186/1756-8935-6-40)
Supplement: Additional file 1 — Model for epigenetic conversions driven by subtle fluctuations of activators and/or silencing factors. (1) All but one of a family of hypothetical varying genes (VG1 to VGN) are maintained in a silenced state. These genes are flanked by a subtelomeric anti-silencing region (STAR) and a chromatin boundary (B). (2) A subtle increase in the abundance of gene activators (green circle) and/or factors that engage STAR (purple circle) and the concomitant passage of replication forks would allow the activators access to the promoters of the VG genes and (3) would predispose all VG loci to derepression. (4) Consequently, during the next stage (re-establishment of silencing), the derepressed VG genes (VG2 to VGN, pink) will compete with the currently active gene (VG1, red). (5) During this stage, a decline in the abundance of activators and STAR-acting factors would aid in the formation of heterochromatin and the limiting activators would be gradually sequestered to a single locus. There is a high probability that the currently active gene (VG1) will be reinstated as the active locus. However, switches to another gene are possible (VGN). The likelihood of such switches, depicted by the width of the arrow, represents the frequency of epigenetic conversions. [file 1756-8935-6-40-S1.pptx]

## Slide 1
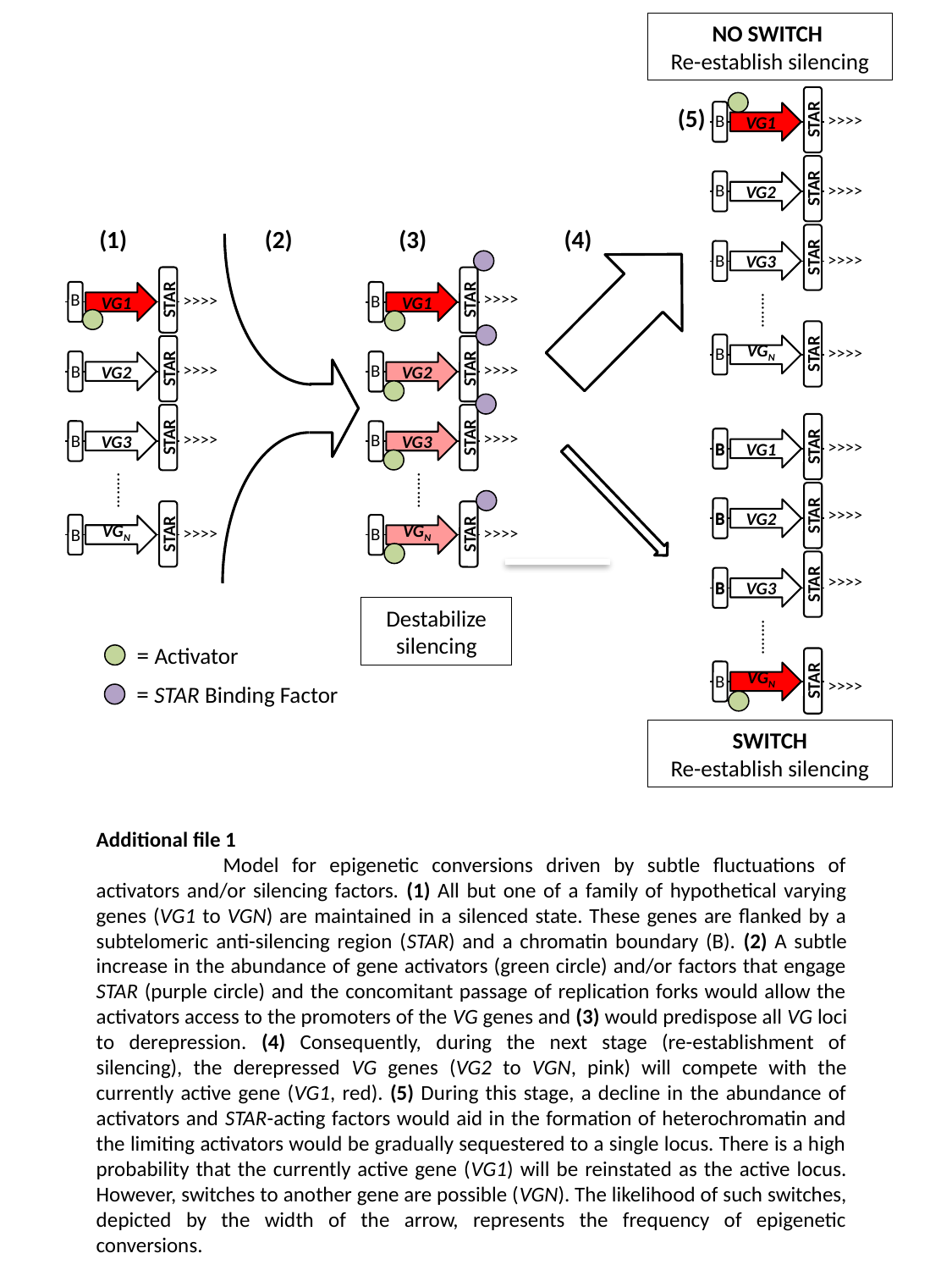

NO SWITCH
Re-establish silencing
VG1
>>>>
STAR
VG2
>>>>
STAR
VG3
>>>>
STAR
........
VGN
>>>>
STAR
B
B
B
B
(5)
(1)
(2)
(4)
(3)
VG1
>>>>
STAR
VG2
>>>>
STAR
VG3
>>>>
STAR
........
VGN
>>>>
STAR
B
B
B
B
>>>>
VG1
STAR
VG2
>>>>
STAR
>>>>
VG3
STAR
........
VGN
>>>>
STAR
B
B
B
B
B
>>>>
VG1
STAR
>>>>
B
VG2
STAR
>>>>
B
VG3
STAR
........
VGN
>>>>
STAR
B
B
B
B
Destabilize silencing
= Activator
= STAR Binding Factor
SWITCH
Re-establish silencing
Additional file 1
	Model for epigenetic conversions driven by subtle fluctuations of activators and/or silencing factors. (1) All but one of a family of hypothetical varying genes (VG1 to VGN) are maintained in a silenced state. These genes are flanked by a subtelomeric anti-silencing region (STAR) and a chromatin boundary (B). (2) A subtle increase in the abundance of gene activators (green circle) and/or factors that engage STAR (purple circle) and the concomitant passage of replication forks would allow the activators access to the promoters of the VG genes and (3) would predispose all VG loci to derepression. (4) Consequently, during the next stage (re-establishment of silencing), the derepressed VG genes (VG2 to VGN, pink) will compete with the currently active gene (VG1, red). (5) During this stage, a decline in the abundance of activators and STAR-acting factors would aid in the formation of heterochromatin and the limiting activators would be gradually sequestered to a single locus. There is a high probability that the currently active gene (VG1) will be reinstated as the active locus. However, switches to another gene are possible (VGN). The likelihood of such switches, depicted by the width of the arrow, represents the frequency of epigenetic conversions.
